# Beyond Sulfation: Integrated Immunohistochemical, Genomic, and Proteomic Profiling Identifies PAPSS1 as a Potential Regulator of Breast Cancer Progression

**DOI:** 10.3390/cancers18193143

**Published:** 2026-09-29

**Authors:** Rabab Ahmed Ahmed Mohammed, Amina Ismaeel, Safa Alshaikh, Aalaa Mubarak, Sajedah Sabt, Samvel Selvam

**Affiliations:** 1 Department of Pathology, College of Medicine and Health Sciences, Arabian Gulf University, Manama 329, Bahrain; aminaaya@agu.edu.bh (A.I.); samvels@agu.edu.bh (S.S.); 2 Department of Pathology, Salmaniya Medical Complex, Manama 329, Bahrain; sshaikh1@hospitals.gov.bh (S.A.); amubarak2@hospitals.gov.bh (A.M.); 3 Department of Oncology, Salmaniya Medical Complex, Manama 329, Bahrain; ssabt@hospitals.gov.bh; 4 Department of Pathology, Faculty of Medicine, Assiut University, Assiut 71515, Egypt

**Keywords:** breast cancer, PAPSS1, clinicopathological features, progression

## Abstract

**Background:** PAPSS1 (3′-Phosphoadenosine 5′-Phosphosulfate Synthase 1) plays a central role in the sulfation process that regulates estrogen activity; however, its pattern of expression and association with clinicopathological features in breast carcinoma (BC) are not fully examined. **Methods:** A total of 150 BC specimens with clinicopathological and 10-year follow-up data were analyzed. PAPSS1 protein expression was assessed using immunohistochemistry (IHC) and correlated with clinicopathological parameters and patient outcome. Transcriptomic and promotor methylation analyses were performed using TCGA-BRCA through UALCAN. Proteomic data was evaluated using the CPTAC dataset, and the gene interaction network was analyzed using STRING. **Results:** IHC demonstrated significantly higher PAPSS1 expression in invasive carcinomas compared with non-invasive carcinoma and with normal mammary acini (*p* < 0.0001). High expression was associated with the presence of vascular invasion (VI) (*p* = 0.003), LN metastasis (*p* = 0.008), and higher PAPSS1 expression in tumor capillaries (*p* < 0.001). CPTAC analyses confirmed significantly elevated PAPSS1 in tumors versus normal tissues (*p* < 0.0001), particularly in stage II and III tumors, triple-negative BC, and basal stromal-related molecular subtypes. The transcriptomic data showed different expression patterns according to tissue type; it is higher in GIT tumors. The opposite was found in lung and pancreatic carcinoma. In BC, transcriptional expression was not different between normal and tumors; it was lower in higher nodal stages and highest in medullary carcinoma and in TP53-mutant tumors. **Conclusions:** PAPSS1 is overexpressed in BC and is associated with VI, LN metastasis, and tumor progression, suggesting a potential role in BC aggressiveness. Further studies are recommended to explore its mechanistic role.

## Simple Summary

Breast cancer progression is influenced by several molecular pathways, including those involved in hormone regulation. PAPSS1 is an enzyme that participates in the sulfation process that can affect estrogen activity and other cellular functions related to tumor behavior; however, its role in breast cancer has not been fully clarified. In this study, we examined PAPSS1 protein expression in breast cancer tissue samples and analyzed its genetic and proteomic features using large public cancer databases. We found that PAPSS1 expression was significantly higher in invasive breast cancer compared with normal breast tissue and was associated with features linked to aggressive disease, including vascular invasion and lymph node metastasis. Higher expression was also observed in triple-negative and advanced-stage tumors. These findings suggest that PAPSS1 may contribute to breast cancer progression and could represent a potential biomarker or therapeutic target for future research.

## 1. Introduction

Breast carcinoma (BC) is the most common malignant tumor in women worldwide [1]. Tremendous research work is ongoing to identify mechanisms underlying its development and progression. Estrogen is a well-known key player in the development and progression of BC. Regulation of estrogen function is largely dependent on estrogen sulfation [2], the process in which a sulfate group is added to a molecule. It is an essential process in the regulation of the activity of several hormones, including estrogen. The sulfation process is catalyzed by a family of enzymes called sulfotransferases [3]. The sulfotransferase enzyme that sulfates estrogen is called SULT1E1 [is known as estrogen sulfotransferase]. SULT1E1 catalyzes the sulfation of active 17β-estradiol (E2), forming the inactive estradiol sulfate in targeted tissues (Figure 1). The inactive estradiol sulfate can be reactivated following desulfation by estrogen sulfatase. The sulfation process then depends upon the sulfate group, which comes from the sulfate donor called “PAPS” [(3′-phosphoadenosine-5′-phosphosulfate)]. Production of PAPS is dependent on the enzyme PAPSS [PAPS—synthesized enzymes) [3], Figure 1.

PAPSS is a multifunctional enzyme responsible for the formation of PAPS, the sole source of sulfate for sulfation in human tissues [4]. Recent evidence suggests that sulfonation may have a role in carcinogenesis and progression of some malignant tumor types; however, knowledge and data about this process in relation to oncology are still minimal. PAPSS has two isoforms, PAPSS1 and PAPSS2. These two isoforms have different functions. By searching the literature, two studies examining PAPSS1 in breast cancer were found; in one study, overexpression of PAPSS1 was found to influence cell growth and apoptosis in malignant breast cancer cell lines [2]. In the other study, it was found that PAPSS1 mRNA level is significantly lower in brain metastasis compared with primary tumors, and this high expression was associated with better overall survival [5]. The clinical outcome of PAPSS expression, either PAPSS1 or PAPSS2, varies highly depending on the specific organ site and the metabolic context of the tumor. In esophageal squamous cell carcinoma (ESCC), PAPSS1 was found to be significantly upregulated and became abundant in tumor tissues, resulting in tumor progression and lymph node (LN) involvement. When *PAPSS1* is knocked down, cell migration and proliferation are significantly suppressed in ESCC cell lines [6]. In colorectal carcinoma, a reduction in PAPSS2 was found to induce damage to the intestinal mucosal barrier, increase intestinal permeability, and bacterial infiltration. Interestingly, sulfation alteration was also found to have a significant influence on the tumor microenvironment, leading to tumor progression and worsening of the patients’ survival [7].

In the current study, PAPSS1 was selected to be explored in BC because previous evidence suggests that it has a more prominent role in BC biology than PAPSS2 [2]. Moreover, very little is known about PAPSS1 in BC in terms of its pattern of expression and its relationship to clinical, histopathological, genetic, and proteomic features. The present study aims to assess: 1—the level and pattern of PAPSS1 protein expression in human BC and its association with clinicopathological tumor features and patients’ outcome, and 2—features of genetic and proteomic changes in PAPSS1 in BC and its network of interaction with other genes using the available genomic and proteomic databases.

## 2. Materials and Methods

### 2.1. Specimens

Histopathological data was identified for 221 primary invasive breast cancer specimens from the archive of the pathology laboratory, Salmaniya Medical Complex (SMC), Kingdom of Bahrain. These specimens were surgically removed from BC patients in the period from January 2016 to December 2019. Data was identified as follows: patient age, tumor histopathological type, grade, size, site, presence of vascular invasion (VI), estrogen receptor (ER) status, progesterone receptor (PR) status, HER-2 status, and status of axillary lymph nodes (LNs). Vascular invasion was identified by examination of all the H&E-stained tissue sections of the BC specimens; no vascular endothelial markers were used.

In this study, and for the purpose of assessing the relationship between alteration of PAPSS1 expression and patient outcomes, patients’ follow-up data for 108 months (range: 1 to 108 months and mean: 73 ± 2.3 months) were identified. Overall survival (OS) was defined as the period from primary surgery until the death of the patient; data for death from breast cancer were available for 164/221 (164 out of 221 patients). The disease-free interval (DFI) was defined as the period from the end of the primary treatment until the occurrence of any locoregional recurrence. Locoregional recurrence is defined as a tumor arising in the treated breast, the chest wall, or in the axillary or internal mammary LNs. Data were available for 169/221 patients. Distant metastasis is identified as secondary tumors appearing at any remote site; data were available for 168/221 patients (Supplementary Figure S1). Any of these events (locoregional, distant metastasis, or death) was scored as an event with censoring of other patients at the time of the last follow-up. Only excisional biopsies that had well-representative tumor tissues were selected for further assessment of PAPSS1 expression (150 blocks in total). All examined specimens were formalin-fixed paraffin-embedded (FFPE) blocks. Methods used in this study were carried out in accordance with national guidelines and regulations. All procedures performed were conducted in accordance with the ethical standards of the institutional and national research committee and with the 1964 Declaration of Helsinki and its later amendments.

### 2.2. Immunohistochemical Staining

One hundred and fifty cases had available, well-representative tissue blocks, and those were retrieved for immunohistochemical (IHC) staining. One tissue section was cut at 4 μm from each tumor block, and the sections were baked at 60 °C for 30 min, dewaxed in xylene, and then rehydrated in a graded series of alcohol ending with deionized water. Sections underwent antigen retrieval using citrate buffer in a microwave for 20 min, then sections were left at room temperature (RT) to cool and then washed in distilled water (DW) for 2 min. The reaction of endogenous peroxidase was blocked, and then sections were rinsed with phosphate-buffered saline (PBS), pH 7.6, twice, 2 min each. Each slide was then incubated with the primary antibody as follows: PAPSS1; Abcam, Cambridge, UK, Rabbit polyclonal Ab, Cat #ab155600 at 1:300 for one hour at room temperature in a humidity chamber. For visualization of the antibody reaction, Leica, Novolink™ Polymer Detection System kit, Product No: RE7280-K, Leica Biosystems Newcastle Ltd., Newcastle upon Tyne, UK., was used according to manufacturer instructions. Sections were washed in DW, counterstained with Myer’s hematoxylin, Thermo Fisher, Waltham, MA, USA, washed again in DW, dehydrated in ascending grades of ethanol alcohol, then cleared in xylene and finally mounted with DPX and cover slipped. Positive control sections of gallbladder sections, as well as negative controls [omission of the primary antibody and substituting it with PBS], were used (Supplementary Figure S2). The PAPSS1 antibody used in this study from Abcam was selected as specific for PAPSS1 based on the manufacturer’s validation data, including Western blot and immunohistochemical validation. The used antibody targets the C-terminal region specific to PAPSS1 and does not have cross-reactivity with PAPSS2. No peptide-blocking experiments were performed in the current study.

### 2.3. Assessment of PAPSS1 Expression

Most tumor sections contain the invasive tumor itself, areas of carcinoma in situ, and normal mammary glands in the surrounding peritumoral areas. PAPSS1 was examined in these areas: (1) normal mammary acini, (2) in non-invasive (in situ) tumor component, (3) in the invasive tumor areas, (4) in the tumoral stroma inflammatory cell infiltrate, and (5) in the vessels located both inside and surrounding tumor tissue.

PAPSS1 expression in the tumoral cells was assessed according to the intensity of positive staining [grade (0) was given for specimens with complete negative staining, grade (1) for weak staining, grade (2) for moderate staining, and grade (3) for strong staining]. The percentage of positive tumor cells was identified for each tumor. Then, the staining grade was multiplied by the % of positive staining to obtain a final PAPSS1 IHC—score for each specimen. The median PAPSS1 H-score (80) was used as the cutoff value to categorize the tumors into two groups. Specimens with a PAPSS1 H-score ≤ 80 were classified as the low-expression group (group A), whereas those with a PAPSS1 H-score > 80 were classified as the high-expression group (group B). The median H-score was selected as an objective, unbiased, and commonly used method to divide a cohort into two groups. PAPSS1 expression in the inflammatory cells in the tumor was identified as either negative or positive. Positive staining for PAPSS1 in tumoral endothelial cells of the capillaries was divided into negative, low, or high expressions according to the intensity of staining compared with the intensity of vessels in the non-tumoral areas outside the tumor. For confirmation of scoring accuracy, ImageJ analysis software (ImageJ 1.46r) was used to assess staining intensity of 20% of sections independently by another investigator blinded to the IHC scores. Both readings were compared, and a Pearson correlation coefficient was computed to evaluate the relationship between the two readings. The results indicated a strong correlation [0.98]. A Pearson correlation coefficient of 0.98 was calculated using the continuous PAPSS1 IHC scores generated independently from ImageJ-assisted image analysis and manual assessment.

### 2.4. Assessment of the Cytomorphological Features of Tumor Cells

Mitosis, degree of tubule formation, and nuclear pleomorphism are the main morphological features according to which tumors are graded. Each feature was assessed in each of the breast cancer sections according to the standard guidelines in the pathological diagnosis of BC [8] as follows: tubule formation was assessed by identifying the proportion occupied by tumor islands showing clear acinar or gland formation or defined tubular structures with a luminal space using a low-power scan of the tumor sections. A tumor in which 75% or more of its area is composed of such structures would score 1 point for gland/tubule formation. A tumor with 75% to 10% of glandular/tumor area would score 2 points. A tumor with less than 10% gland/tubule formation would score 3 points. Nuclear pleomorphism was assessed as follows: minimal nuclear variation in size and shape (<1.5 times the size of benign epithelial cell nuclei), even chromatin, and inapparent to inconspicuous nucleoli was identified as score 1. Moderate nuclear variation in size and shape (1.5–2 times the size of benign epithelial cell nuclei), typically visible but with small nucleoli, was identified as score 2. Marked nuclear variation in size and shape (>2 times the size of benign epithelial cell nuclei), and often prominent nucleoli were identified as score 3. Mitosis was assessed by counting the number of mitotic figures in the most active area in 10 high-power fields using the microscope objective lens (×40), which is comparable with the field diameter of 0.44 mm. Mitotic count between 0 and 5 takes score 1, between 6 and 11 takes score 2, and >11 mitotic figures takes score 3.

### 2.5. Genetic, Epigenetic, and Proteomic Analysis

The second part of this research was an in silico study conducted using the publicly available datasets from the Cancer Genomic Atlas (TCGA) accessed via the UALCAN interactive web resource [access date: 1 April 2026, https://ualcan.path.uab.edu/] [9,10]. Breast carcinoma datasets (TCGA-BRCA) were utilized to evaluate the expression and molecular characteristics of *PAPSS1* across tumor and normal breast tissue (number of tumor samples = 1097 and normal samples n = 114). The following analysis was conducted: [A] the transcriptional expression levels of PAPSS1 were analyzed using RNA-sequencing data (Illumina HiSeq platform, Illumina, Inc., San Diego, CA, USA). Expression values were presented as transcripts per million (TPM) and compared between primary and normal samples. Subgroup analysis was performed based on clinicopathological parameters, including tumor stage, molecular subtypes, nodal stage, and tumor stage. The TCGA-BRCA specimens included in the analysis in this study were only those that had available RNA-sequencing data. All pathological types of BC were included. Cases lacking expression data or relevant clinicopathological annotations were excluded. PAPSS1 mRNA expression was evaluated according to tumor stage, molecular subtype, nodal status, TP53 mutation status, and patient survival. Statistical analysis was conducted using Student’s *t*-test as implemented within the UALCAN platform; [B] protein expression was evaluated using data derived from the Clinical Proteomic Tumor Analysis Consortium (CPTAC) integrated within the UALCAN portal (number of tumor samples = 125 and number of normal samples = 18). Data was presented as Z-scores normalized to standard deviations from the median expression of normal samples; [C] promotor methylation levels of the *PAPSS1* gene were assessed using TCGA DNA methylation data (number of tumor samples = 885 and number of normal samples = 97). Beta values (ranging from 0 to 1) were used to quantify methylation status, where higher values indicate increased methylation; and [D] to explore the functional interaction network of *PAPSS1*, protein–protein interaction (PPI) analysis was performed using the STRING database [https://string-db.org/] [11], access day: 21 April 2026. *PAPSS1* was added in the search term, and homo sapiens was selected as the organism. The schematic pathway diagram was generated with the assistance of an artificial intelligence-based image generation tool (OpenAi DALL.E) and subsequently reviewed and curated by the authors for scientific accuracy.

### 2.6. Statistical Analysis

Specimens were subdivided into two classes according to the level of expression of PAPSS1, using the median value to form a group with low PAPSS1 expression and a group with high PAPSS1 expression. Histopathological features were categorized into dichotomous variables. Chi-square and 2 × 2 tables were used to assess a significant association between clinicopathological features of tumors and the expression of PAPSS1. Means of PAPSS1 in normal, non-invasive, and invasive tumors were compared using ANOVA. Two-tailed *p*-values of less than 0.05 were considered significant. Survival analysis was conducted using the log-rank test and Kaplan–Meier survival curves. Statistical analyses were performed using IBM SPSS Statistics for Windows, Version 26 (IBM Corp., Armonk, NY, USA). 

## 3. Results

### 3.1. Clinicopathological Features of Specimens

A cohort of primary breast carcinomas (221 specimens) was identified. Patients ranged in age from 28 to 89 years old, with a mean of (51 ± 0.8) years; 107 patients (48%) were younger than 50 years. Tumor sizes ranged from 0.1 to 13.5 cm (mean 3.4 ± 0.18). Most of the patients were of stage II and III [102 patients (74%) and 74 (35%), respectively], with 126 patients (65%) having lymph node positivity. Ten (6%) patients developed local recurrence, and 52 (31%) developed metastasis. Only specimens that have available tissue blocks of good quality were selected for PAPSS1 staining (150 blocks from 150 cases).

### 3.2. PAPSS1 Pattern and Level of Expression by IHC

PAPSS1 positive staining was detected in the cytoplasm of tumor cells (Figure 2A,B), in the vascular endothelium of tumor vessels inside and around tumor tissue (Figure 2C), in addition to occasional staining in the inflammatory cells infiltrating the tumoral microenvironment (Figure 2C).

The level of PAPSS1 expression was found to be heterogeneous among specimens, ranging from negative to strong staining (Figure 3). IHC score for PAPSS1 ranged from 0 to 300, mean 87 ± 6.4, median 80 ± 70. PAPSS1 expression was significantly higher in invasive tumor tissues [mean: 92 ± 5.6 (SD; 69.39)] than in the in situ tumor tissues [mean: 30 ± 3.9 (SD; 33.5)] and normal peritumoral tissues [mean: 7.7 ± 0.8 (SD; 9.4)], *p* < 0.0001, Figure 2D.

### 3.3. Association Between PAPSS1 Expression by IHC with Clinicopathological Features and Patient Survival

PAPSS1-high expression showed a significant association with the presence of VI; 32 (55%) of the PAPSS1-high group showed positive VI compared with 27 (30%) of VI-negative specimens (*p* = 0.003, OR 2.8, 95% CI: 1.4–5.6 and Carmer’s V: 0.248). A Similar association was found in relation to the presence of LN metastasis (*p* = 0.008, OR 2.9. 95% CI: 1.3–6.7, and Cramer’s V: 0.229). All these significant associations retained significance after adjustment for multiple comparisons using the Benferroni test. Forty-six specimens (82%) of the PAPSS1-high group showed malignant deposits in one or more axillary LNs compared to 61% of the PAPSS1-low group. PAPSS1 high expression in the tumor cells was significantly associated with high PAPSS1 expression in the vascular endothelium within the tumor area, *p* < 0.000. No significant association was detected with ER status (*p* = 0.734) or with PR status (*p* = 0.182). No significant association was observed in relation to tumor grade, stage, or HER-2 status. Table 1 provides more details about the relationship between PAPSS1 expression and clinicopathological features.

An assessment of the relationship between the expression of PAPSS1 and the cytomorphological features of tumor cells, namely the degree of tubule formation, mitosis, and nuclear pleomorphism, was conducted. High PAPSS1 expression was found significantly associated with low mitosis (*p* = 0.036); however, this significance was lost after adjustment for multiple comparisons using the Benferroni test and therefore should be taken cautiously. No significant association was found with tubule formation (*p* = 0.305) or with nuclear pleomorphism (*p* = 0.290).

Assessment of the association between PAPSS1 expression and the occurrence of recurrence or development of distant metastasis was conducted. No significant difference was detected in relation to the outcome. Two patients (4.2%) of the PAPSS1-high-expressing specimens developed recurrence compared with five patients (6.5%) of the PAPSS1-low-expressing tumors, *p* = 0.583. Seventeen patients (36%) of the PAPSS1-high-expressing group developed distant metastasis compared with 22 patients (30%) of the PAPSS1-low-expressing group, *p* = 0.475. Kaplan–Meier survival curves are seen in Figure 4.

### 3.4. Genetic and Proteomic Features of PAPSS1 in Breast Carcinoma

- PAPSS1 proteomic:

Based on data on the CPTAC dataset, PAPSS1 protein expression was significantly higher in tumors compared with normal tissues (*p* < 0.0001), Figure 5A. It was significantly higher in stage 2 and stage 3 tumors, *p* < 0.0001 and *p* = 0.005, respectively, Figure 5B. The expression was higher in triple-negative (TN) phenotype (*p* = 0.09), compared to all other types, but was not different between HER-2 and luminal types, Figure 5C. It was higher in invasive ductal carcinoma (IDC) versus normal tissues (*p* < 0.0001) and versus invasive lobular carcinoma (ILC) (*p* = 0.002). It was interesting to find that the PAPSS1 protein is significantly higher in tumors that are classified as K4, K7, and K10 classes of breast carcinoma, as in Figure 5D. These classes are molecular subtypes identified through mass spectrometry, each with unique biological pathways [12]. Class 4 is known as basal-like breast cancer with characteristic overexpression of YAP1 and MYC targets; K7 class is known as stromal-related tumors with overexpression of collagen VI proteins; Wnt and Notch pathway signatures, and K10 class are tumors with overexpression of endoplasmic reticulum-related proteins and steroid biosynthesis pathway proteins, *p* = 0.004. This finding may explain why we found an association with vascular invasion in the IHC cohort. No significant association with survival was seen (*p* = 0.18).

- Transcriptional expression of PAPSS1

In the breast, PAPSS1 transcriptional expression was not significantly different between tumors compared with normal tissues, *p* = 0.327. It was interesting to find that the PAPSS1 mRNA expression pattern is different according to tumor tissue type. It was higher in the tumors than in normal tissues in the tumors of the thyroid and GIT (esophagus, stomach, colon, rectum, liver, and cholangiocarcinoma), while the opposite was observed in tumors of the breast, kidney, lung, and pancreas, where it was lower in the tumors compared with the normal tissues (Figure 6). In breast carcinoma, mRNA PAPSS1 expression was highest in the TN phenotype compared with the luminal and HER-2 types. It was higher in the luminal types compared with HER-2, *p* < 0.001. Its expression was highest in the medullary carcinoma (*p* = 0.005) and was higher in infiltrating duct carcinoma (IDC) than in infiltrating lobular carcinoma (ILC) (*p* < 0.0001). In relation to the LN stages, PAPSS1 mRNA expression was lower in patients having more than 3 + LN compared with LN-negative or patients with less than 3 + LNs. It was also significantly higher in breast cancers that have TP53 mutant status than in non-mutant (*p* < 0.0001).

- Analysis of PAPSS1 protein–protein interaction network

Based on protein–protein interaction analysis using the STRING database, PAPSS1 was found to be closely related in a network to the following proteins: (1) PAPSS2 (a paralog of PAPSS1), (2) BPNT1 and IMPD1 (involved in sulfation metabolism), (3) SLC26A2 (is a sulfate transporter), (4) SUOX (converts sulfite to sulfate), (5) FLAD1 (involved in FDA biosynthesis), and (6) EEF1A1, EEF1A2, GSPT1, and HBS1L; these four genes are all involved in the translation elongation/termination, which reflects high protein synthesis demand. Figure 7 and Figure 8 show the PASS1 protein–protein interaction network. Both Figure 7 and Figure 8 are derived from STRING database-predicted and literature-supported protein–protein interaction networks and do not represent experimental validation performed within the present study.

## 4. Discussion

PAPSS is the enzyme that synthesizes PAPS (3′-phosphoadenosine-5′-phosphosulfate), the exclusive source of sulfate for the sulfation process in human tissues. Sulfation is a crucial process in cell biology that regulates the functionality of secreted proteins, hormones, and signaling molecules. Two principal isoforms of PAPSS exist: PAPSS1 and PAPSS2. Their distribution within the tissues and intracellularly is distinct. This study consists of two components: the first component evaluated the pattern and level of PAPSS1 protein expression in 150 primary breast cancer specimens through immunohistochemistry, while the second component involved analysis of genetic and proteomic data pertaining to *PAPSS1* from publicly accessible datasets, including TCGA, CPRAC, and STRING.

In the BC specimens, the evaluation of PAPSS1 protein expression with IHC revealed significantly elevated levels in invasive tumor tissue compared to non-invasive tumors and normal tissues. Identical results were observed in the examination of proteomic data from the CPTAC dataset. Comparable results were also documented in prior research on breast cancer and endometrial carcinoma [2]. The initial alteration in PAPSS1 expression may suggest its involvement in carcinogenesis, a theory corroborated by evidence that sulfonation can produce reactive electrophiles capable of binding to DNA, potentially triggering mutagenic or carcinogenic responses [13,14]. The markedly elevated PAPSS1 in breast cancer tissues was observed at the protein level, but not at the mRNA level. It is acknowledged that mRNA and protein abundance cannot serve as direct proxies for each other; protein levels are affected by translational and post-translational modifications [15]. Cancers are characterized by modified translational processes and dysregulated protein turnover and signaling pathways that affect protein synthesis and stability [16].

For different molecular phenotypes of BC, PAPSS1 expression was found significantly higher in TNBC compared with other types, as was observed in the CPTAC proteomic analysis, whereas no statistically significant association with TN status was identified in our IHC cohort. This discrepancy may be a potential consequence of sample size differences, biological heterogeneity, and platform-specific effects.

In our specimens, PAPSS1 positive staining was detected in the cytoplasm of the tumor cell with perinuclear concentration. Occasional nuclear staining was seen. The antibody used in this study is an immunogen corresponding to the recombinant fragment protein within human PAPSS1 aa 300 to the C-terminus (Abcam catalog # AB155600). This antibody recognizes epitopes at the C-terminal of the AB. PAPSS1 antibodies, where nuclear staining was detected, are monoclonal antibodies raised against amino acids 288–329 mapping within an internal region of PAPSS1 of human origin. This kind of antibody is more epitope-specific and therefore more restricted. PAPSS1 was originally described to have nuclear localization [17]; further studies found that PAPS synthase isoforms are mobile enzymes capable of shuffling and executing their function in the cytoplasm as well as in the nucleus [18]. We think the difference in localization of positive staining may be due to differences in antibodies used on one side and may also be due to protein changes, such as truncation, cleavage of PAPSS1, or post-translational modifications. All these changes will influence staining sensitivity and the detection of truncated protein forms. Some studies reported mixed nuclear and cytoplasmic localization of PAPSS1 protein [2], while others showed mainly nuclear [19].

The first portion of this analysis on BC tissues found a significant correlation between high PAPSS1 expression and VI and LN metastasis. In a previous gene expression profiling analysis, increased PAPSS1mRAN expression in breast cancer was related to VI [20]. VI is complex and involves tumoral cell interaction [21,22]. VI discovery in BC has long been a strong predictive marker for poorer outcomes [23,24,25,26]. Malignant tumor cells can create new vessels and invade and disseminate via them. Several processes are needed for vascular invasion. Some clinical and molecular tumor features of VI were found [21,22,26]; however, the exact mechanism is still unknown. Tumor cells must invade the extracellular matrix (ECM), attach to endothelial cells to enter vascular channels, evade the antitumoral immune response, and then attach again to leave the vascular stream to settle in new tissue sites [27]. Proteoglycans like chondroitin sulfate (CS) at the cell surface and the ECM are important proteins. Melanoma, prostate, breast carcinoma, and other cancers express CS in their blood vessels and stroma. They are known to aid tumor development and invasion [28]. PAPS post-translationally sulfates proteoglycans (PGs) like heparan sulfate, regulating cell growth, motility, and invasion [29]. However, how PAPSS enzyme activity and PAPS availability affect heparan sulfate proteoglycans (HSPG) structure and function is unclear.

It was noteworthy in the present study to observe positive expression of PAPSS1 in the endothelial cells of the tumoral vessels, which was significantly higher than that expressed in the normal vessels in the peritumoral area. By searching the literature, we found that PAPSS1 is expressed in the high endothelial venules (HEVs). The endothelium of the HEVs is different from the endothelium in other body vessels in that several ligands and receptors, such as L-selectin, P-selectin, and CXCL9, become highly expressed on the endothelium in certain conditions, such as inflammation, to allow lymphocytes to exit from the vessels towards the surrounding tissue (homing of lymphocytes) [30]. Cancers are known to adapt normal physiological mechanisms to evade immune reaction, to move, to migrate, and to survive. The molecular mechanism of cancer cell lymphatic metastasis shares similar mechanisms to those of lymphocyte homing [31]. In an experimental study, sulfation of the sialomucins (GlyCam-1 and CD34) on HEV by PAPS is essential for high-affinity recognition by the L-selectin on the lymphocytes. Inhibition of sulfation was found to suppress this binding [31,32]. Based on the findings of this study, coupled with the findings from the Hemmerich study, we hypothesize that the presence of PAPSS1 in the endothelial cells may have a similar effect to its expression in HEVs in the lymphoid organs. Increased PAPSS1 in these endothelial cells may influence the binding affinity with other adhesion molecules, such as L-selectin, P-selectin, or CXCL9, which are all well known to play a central role in the ability of tumor cell invasion. According to CPTAC proteomic data, K4, K7, and K10 breast tumors have greater PAPSS1 protein expression. Mass-spectrometry proteome profiling identified ten breast cancer classes from K1 to K10 with distinct protein characteristics and pathways. K4 is a basal-like breast carcinoma with YAP1 and MYC overexpression. Recent research has linked YAP, an oncogene, to malignant transformation and progression [33]. Figure 5C shows that triple-negative breast tumors expressed PAPSS1 more than luminal and HER-2 phenotypes using proteomic analysis. Most histopathological and molecular characteristics of basal and TN phenotypes are known. High PAPSS1 levels in the TN phenotype may sustain the cellular sulfate pool needed for ECM remodeling and signaling, contributing to its high invasiveness. Invasive cells rely on the sulfation of proteo-glycans like versican and heparan sulfate, which allow malignant cells to break through the primary tumor and migrate. This may explain why our IHC evaluation and CPTAC data showed increased nodal status and advanced tumor stages for high protein PAPSS1. The current investigation demonstrated no significant correlation between PAPSS1 expression and tumor size or grade. The correlation between PAPSS1 expression and patient survival revealed that changing PAPSS1 expression did not correlate with patient outcomes, as evidenced by both the IHC component of this investigation and the analysis of the TCGA and CPTAC datasets. PAPSS1 may play a role in the biology of tumor cells in breast cancer, but without prognostic relevance. This may be due to the small number of cases in this study and the heterogeneity of BC cases. Beyond BC, studies in ovarian and non-small cell lung cancers have demonstrated that low PAPSS1 expression is associated with increased sensitivity to platinum-based chemotherapies, such as cisplatin, and improved survival outcomes. Although there is no strong evidence that PAPSS1 is associated with the outcome in BC, the findings in the present study suggest that PAPSS1 expression levels might be associated with tumor progression by enhancing vascular invasion, which warrants further experimental investigation in BC cell lines and in larger cohorts of BC.

At the start of this study, we hypothesized that PAPSS1 may be associated with BC progression through its effect on estrogen functional activity, as mentioned earlier in the introduction and illustrated in Figure 1. However, the analysis showed no significant association with the hormonal status of BC or with the features that are commonly seen in tumors with high estrogen activity, such as tumor size and grade. This does not mean that PAPSS1 has no effect on estrogen activity, because we do not know the estrogen activity status in the examined specimens, and we did not perform functional experimental studies. However, the observation of the high association with VI may indicate an additional possible role of PAPSS1 beyond estrogen sulfation.

In the second portion of the study, it was interesting to find that high expression of PAPSS1 assessed by IHC was associated with less mitosis in breast cancer specimens. This may be explained by the fact that high PAPSS1 will induce more estrogen sulfation and therefore more estrogen inactivation with subsequent less proliferation. In a recent study, Xu and colleagues demonstrated that overexpression of PAPSS1 can block estrogen-stimulated cell proliferation in MCF-7 breast cancer cells while promoting apoptosis through upregulation of the pro-apoptotic gene bax [2]. This observation was further confirmed by the finding that a reduction in PAPSS1 resulted in increased cell proliferation in esophageal carcinoma and promotion of cancer cell migration [19]. This observation should be interpreted with caution because the statistical significance of low mitosis was lost when adjusted for multiple comparisons. Further studies involving larger cohorts of BC specimens are warranted to determine whether PAPSS1 expression is truly associated with mitosis as a histological and biological characteristic in BC.

PAPSS1, PAPSS2, BPNT1, and IMPAD1 form a sulfate activation and recycling network that regulates PAPS production and turnover, according to protein–protein interaction analysis. The association between SUOX and SLC6A2 emphasizes upstream sulfur metabolism. PAPSS1 interacted closely with EEF1A1, EEF1A2, GSPT1, and HBS1L. These four genes are involved in translation elongation/termination, indicating significant protein synthesis. This collection of four genes is necessary for protein synthesis but also involved in cancer progression, cell death, regulation, and stress response. A recent cancer target, GSPT1, is ubiquitous and elevated in malignancies that regulate the cell cycle [34].

In summary, the results of this study found that expression of PAPSS1 is significantly different among tumors according to organ/tissue of origin, protein level, transcriptome level, and according to cellular localization, with evidence for a strong link with tumor invasiveness and proliferation. PAPSS1 high protein expression is associated with less proliferation on one hand, but with higher invasiveness and promotion of tumor progression on the other hand. It is usually appreciated that highly proliferating tumors are those that are more invasive [35], which is not the case in our findings. Taken together, these results may reflect a moonlighting function of PAPSS1. “Moonlighting” function is a term used in biology when a single protein performs two completely different roles in the cell. The primary function is known as the day-job or the canonical function, which is the well-known function of the protein, while the secondary function is known as the night-job or the noncanonical, often unrelated function [36]. This phenomenon can be explained by three main factors. First is the location, as cytoplasmic location may differ from the nuclear location. Some proteins may be active in a location and hyperactive or “off” in another. Second would be binding to a partner protein. The last is cell stress, when the cell is under certain stress or starving, the protein may abandon its main function and shift to help cell survival or induction of cell death (apoptosis). Malignant tumor environment is a complex dynamic medium [37] where thousands of molecules and cytokines are interacting, shaping the overall cellular biological characteristics of the cell.

Some limitations of this study should be acknowledged. First, the immune histochemical analysis was conducted in a single retrospective cohort from one institution, which may limit the generalizability of the findings to broader patient populations. Second, although the study included a relatively reasonable number of BC cases with long-term follow-up data, the distribution of some histological and molecular subtypes was uneven, potentially reducing the statistical power of subgroup analysis. Third, PAPSS1 expression was assessed primarily at the protein level using immunohistochemistry. No peptide-blocking experiments were performed in the current study to show the specificity of the PAPSS1 reaction, and were dependent on the manufacturer’s validation of specificity. In addition, the bioinformatics analysis was based on publicly available datasets, which comprise independent patient cohorts with different sample compositions and analytical platforms. This may partially explain the observed discrepancies between protein and transcriptomic expression patterns, particularly the lack of significant difference in PAPSS1 mRNA expression between normal breast tissue and breast carcinoma, despite the marked protein overexpression detected by immunohistochemistry and the proteomic analysis. Furthermore, the study is observational in nature and therefore cannot establish a causal relationship between PAPSS1 expression as a biomarker and BC progression. Functional experiments investigating the molecular mechanisms through which PAPSS1 influences tumor aggressiveness, vascular invasion, and metastatic potential were beyond the scope of the present work; however, it is mandatory to find the differences in biological effect between cytoplasmic and nuclear localization and the exact mechanism by which PAPSS1 influences BC progression.

Despite these limitations, the integration between clinicopathological, immunohistochemical, transcriptomic, proteomic, and network analysis done in this study provides a comprehensive evaluation of the profile of PAPSS1 expression in BC and opens the door for exploring in depth this molecule in the biology of BC and other cancer types.

## 5. Conclusions

PAPSS1 showed a highly significant association with the presence of vascular invasion in breast cancer and nodal metastasis, suggesting a significant role in the induction of invasion and mobility of breast cancer cells. While specific associations with tumor grade, stage, or receptor status require further elucidation, the differential expression patterns suggest that PAPSS1 could serve as a biomarker for disease progression. Future studies focusing on the mechanistic pathways involving PAPSS1 and its interactions with other molecular players in breast cancer are recommended.

## Figures and Tables

**Figure 1:**
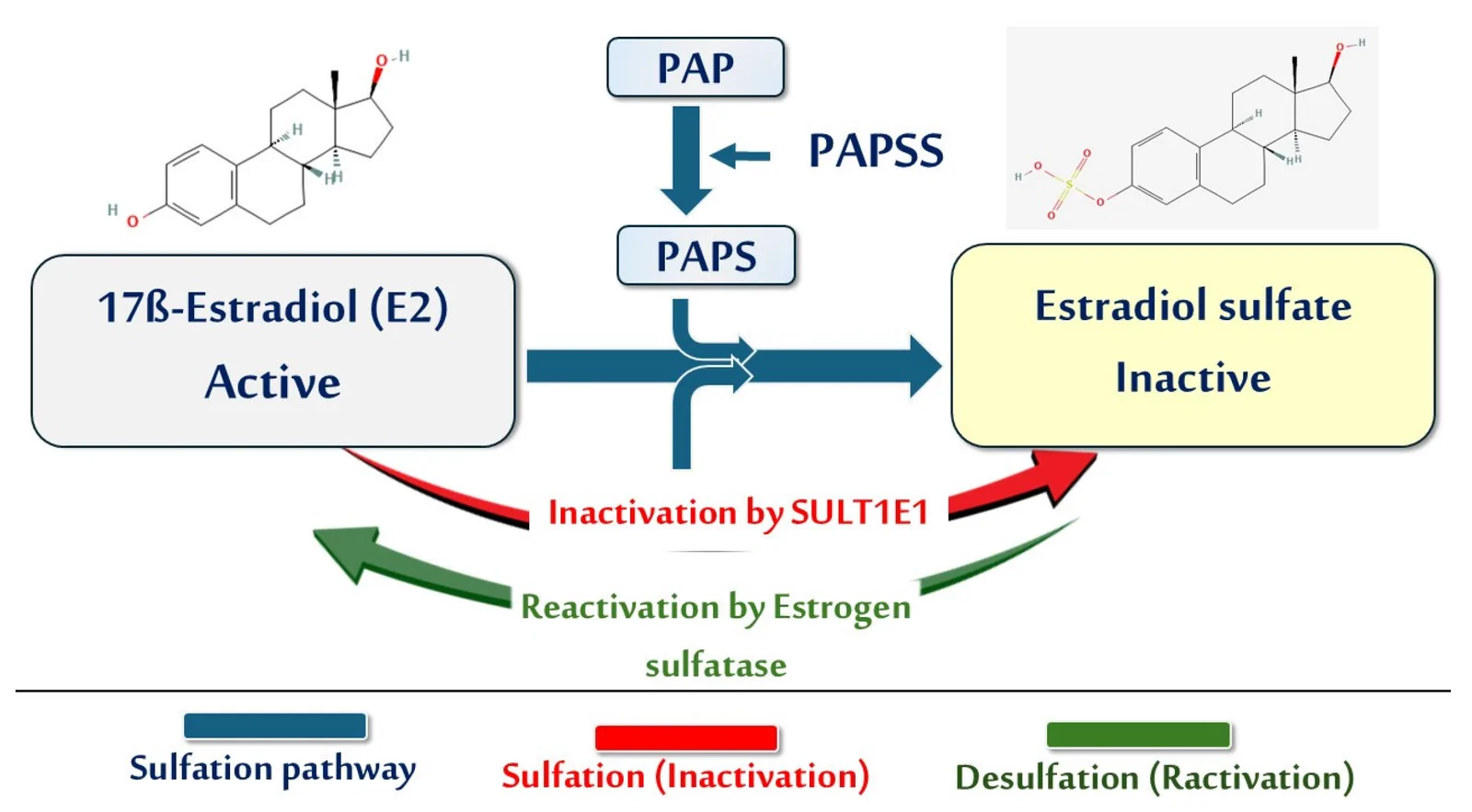
Illustration for the role of PAPSS1 in the regulation of estrogen activation.

**Figure 2:**
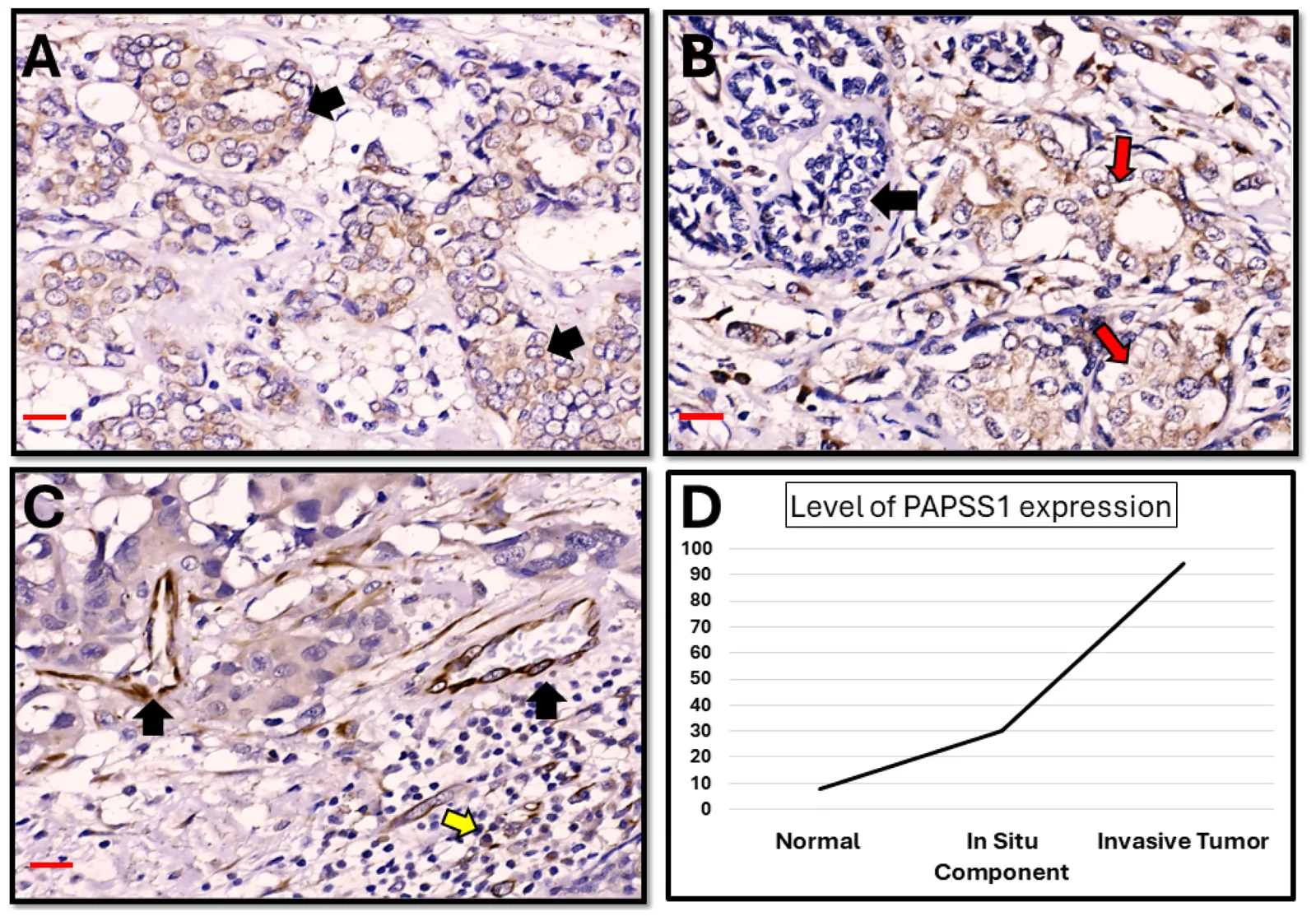
Pattern of PAPSS1 expression in breast carcinoma. (**A**) Invasive breast carcinoma of No Specific Type (NST) showed positive staining for PAPSS1 in the invasive component of the tumor tissue (×400), (black arrows). (**B**) PAPSS1 staining was negative in the normal mammary ducts (black arrow) compared to the malignant tissue (red arrow) (×400). (**C**) Positive staining for PAPSS1 was also observed in the endothelial cells of the tumoral blood capillaries (black arrows) and occasionally in the inflammatory cells (yellow arrow) (×400). The red scale bar is 20 μm. (**D**) Comparison between means of PAPSS1 expression in normal, in situ, and invasive tumor components.

**Figure 3:**
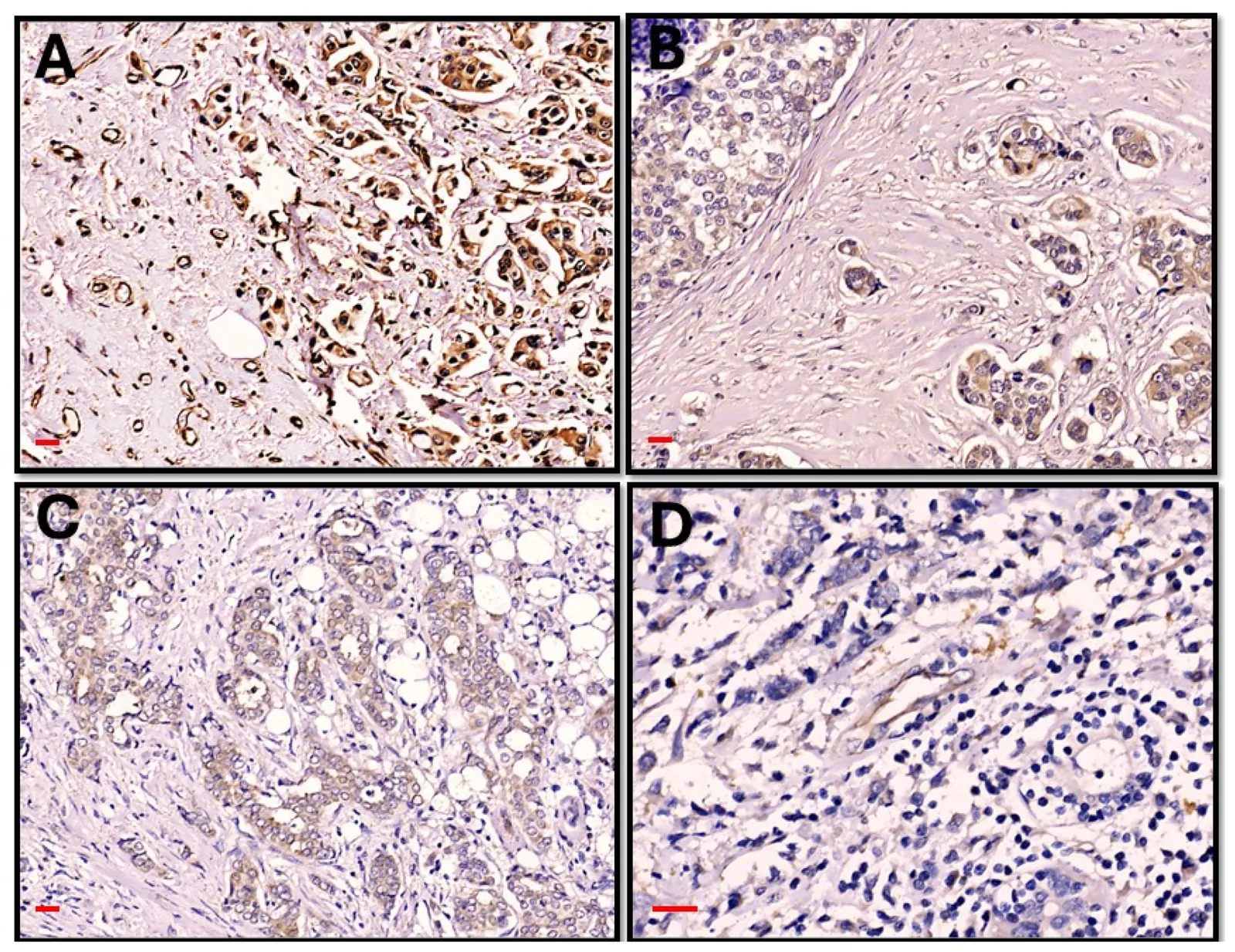
Grades of PAPSS1 expression in breast carcinoma. (**A**) Invasive breast carcinoma grade-3 showed strong staining for PAPSS1 (×200). (**B**) PAPSS1 staining was low in the in situ components (**upper left**) compared to moderate staining in the grade-2 invasive tumor component (**lower right**) (×200). (**C**) Moderate positive staining for PAPSS1 in a grade-1 BC (×200). (**D**) Negative staining for PAPSS1 in the invasive grade-3 BC (in comparison with positive staining in the vascular endothelial cells at the middle) (×400). The red scale bar is 20 μm.

**Figure 4:**
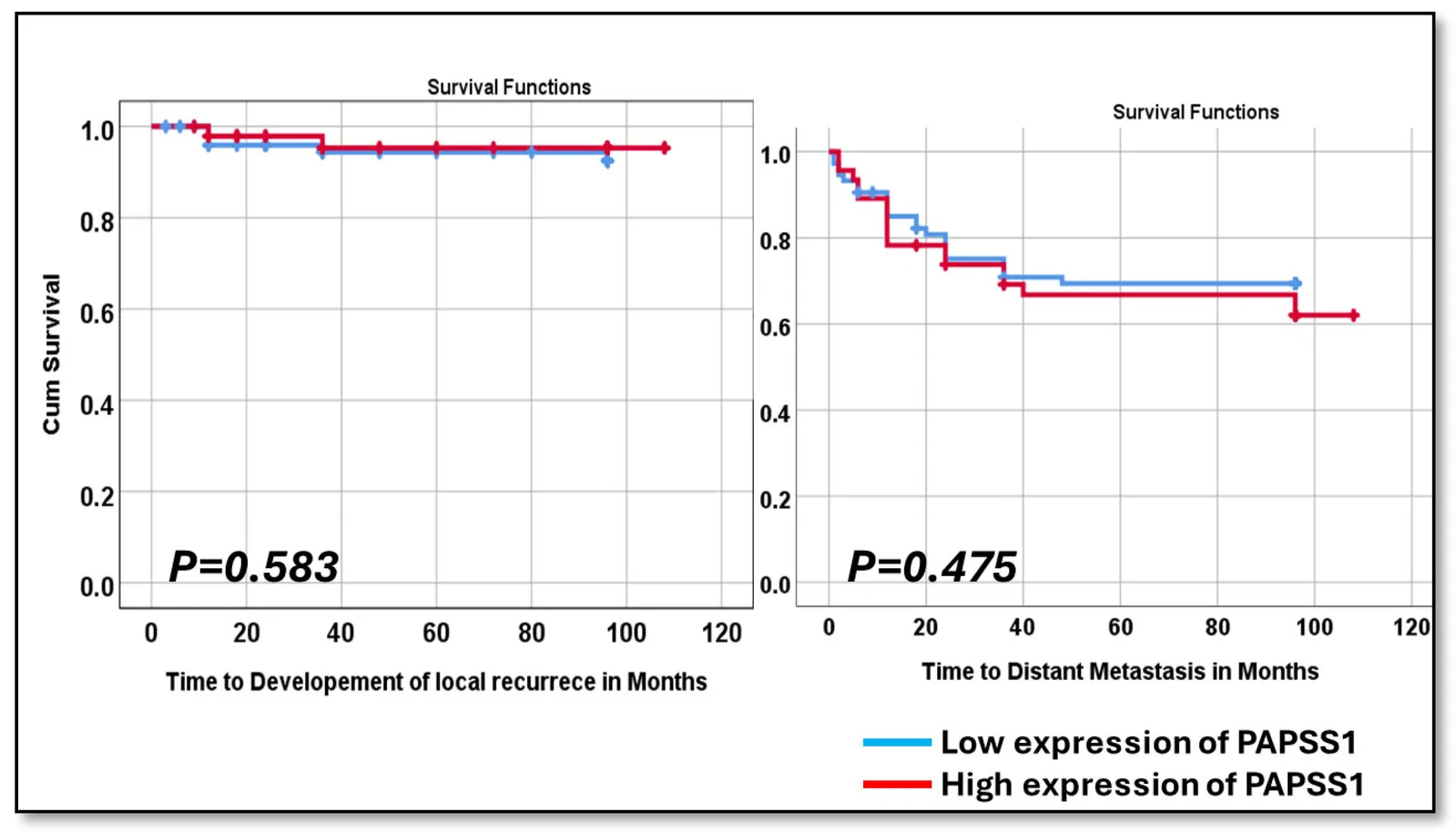
Survival analysis for PAPSS1 expression. Kaplan–Meier curves showing no significant association with the occurrence of recurrence (**left**) or with the development of distant metastasis (**right**).

**Figure 5:**
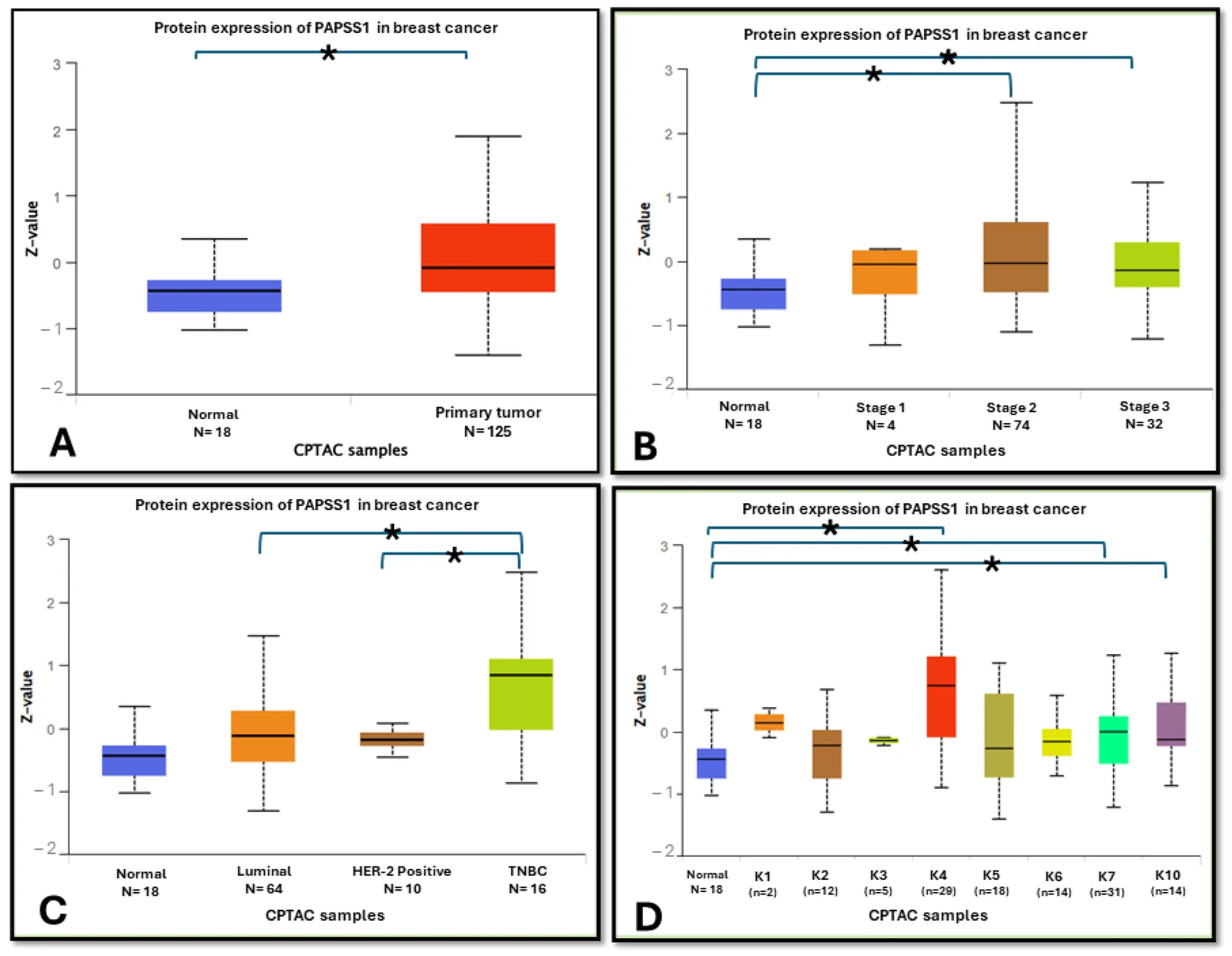
Box-and-whisker plot illustrating PAPSS1 protein expression (Z-score) in breast carcinoma across different clinical groups using the CPTAC data accessed via ULCAN. (**A**) PAPSS1 protein expression is higher in tumors than in normal tissues. (**B**) PAPSS1 expression is progressively increased, particularly in stage II, which exhibits the highest median and widest distribution, including a maximum Z-score of 2.48-, and stage III. (**C**) The expression was highest in the TNBC phenotype compared with both luminal and HER-2 types. (**D**) The expression was also the highest in K4 types (which are known as stromal-related tumors). * indicates statistically significant differences.

**Figure 6:**
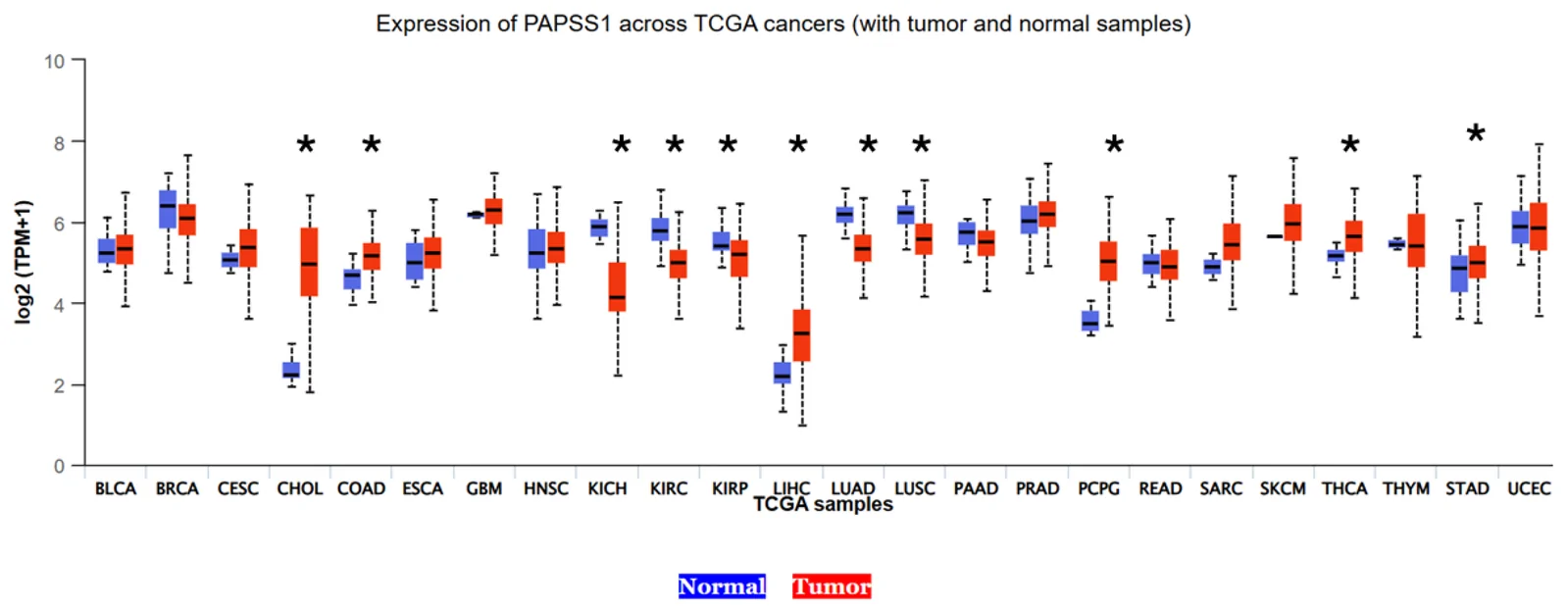
Box-and-whisker plot illustrating PAPSS1 transcriptional expression—measured as transcripts per million (TPM) values-in different tumors according to body tissue types. Red boxes for tumors, blue boxes are normal tissues. Stars (*) indicate statistical significance. BLCA: bladder urothelial carcinoma, BRCA: breast carcinoma, CESC: cervical squamous cell carcinoma and endocervical carcinoma, CHOL: cholangiocarcinoma (* *p* < 0.0001), COAD: colon adenocarcinoma (* *p* < 0.0001), ESCA: eosophageal carcinoma, GBM: Glioblastoma Multiforme, HNSC: head and neck squamous cell carcinoma, KICH: Kidney Chromophobe (* *p* < 0.0001), KIRC: Kidney Renal Cell carcinoma (* *p* < 0.0001), KIRP: Kidney Renal Papillary cell carcinoma (* *p* < 0.0001), LIHC: liver hepatocellular carcinoma (* *p* < 0.0001), LUAD: lung adenocarcinoma (* *p* < 0.0001), LUSC: lung squamous cell carcinoma (* *p* < 0.0001), PAAD: Pancreatic Adenocarcinoma, PRAD: Prostatic Adenocarcinoma, PCPG: Pheochromocytoma and Paragalglioma (* *p* = 0.04), READ: Rectum Adenocarcinoma, SARC: Sarcoma, SKCM: Skin Cutaneous Melanoma, STAD: Stomach Adenocarcinoma (* *p* = 0.004), THCA: Thyroid Carcinoma (* *p* < 0.0001), THYM: Thymoma, UCEC: Uterine corpus endometrial carcinoma.

**Figure 7:**
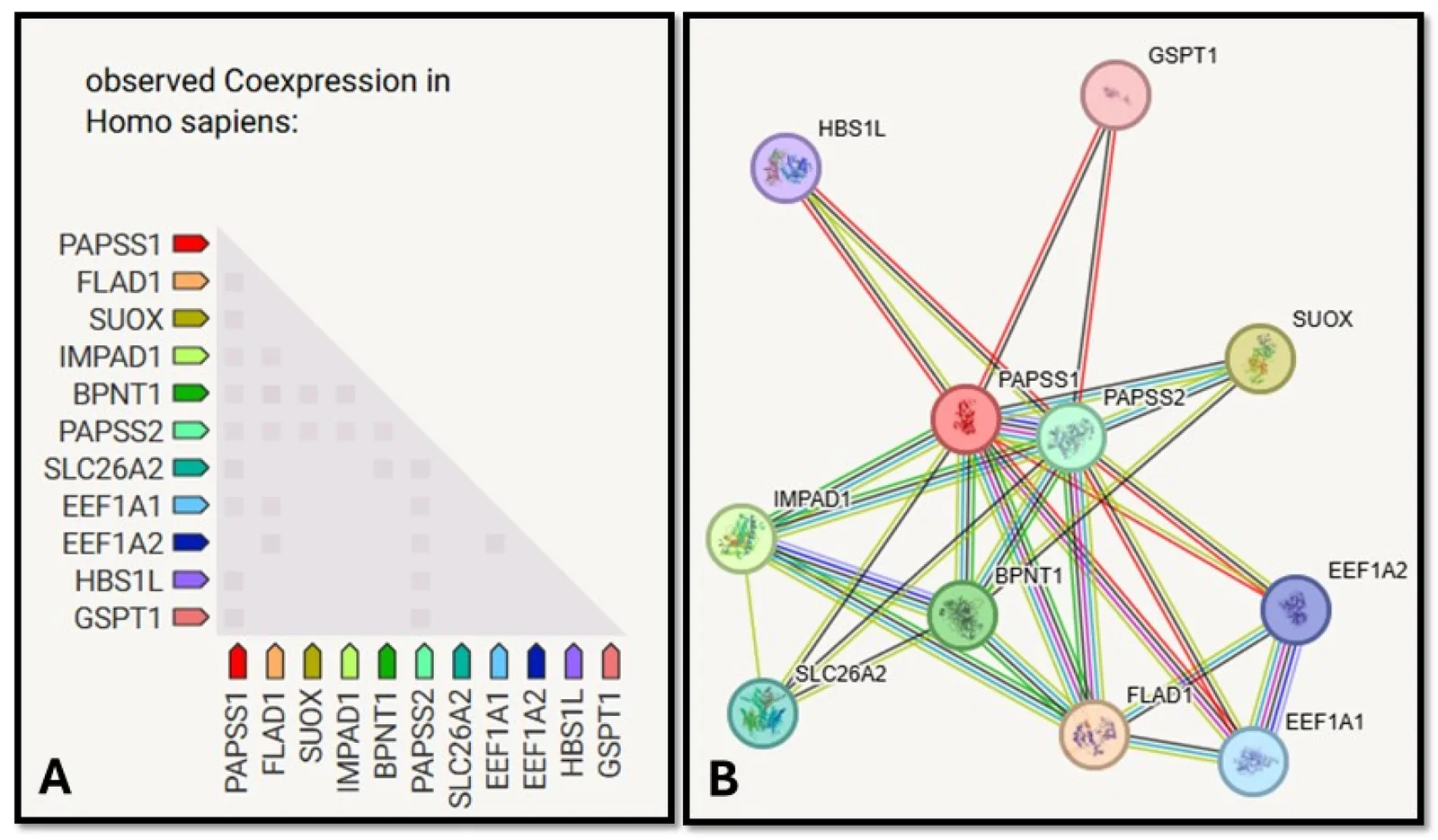
Genes closely related to PAPSS1. (**A**) PAPSS1 is a closely related gene and gene network. Nodes represent the proteins, edges represent connections. Black line: co-expression, blue line: co-occurrence, red line: gene fusion, green line: gene neighborhood (**B**) Diagram showing the predicted functional partners with PAPSS1. It is important to note that these closely related genes are STRING-derived networks which integrate information from multiple evidence sources, including co-expression, curated databases, text mining, and predicted associations, and therefore do not necessarily represent direct physical interactions. Experimental validation is mandatory.

**Figure 8:**
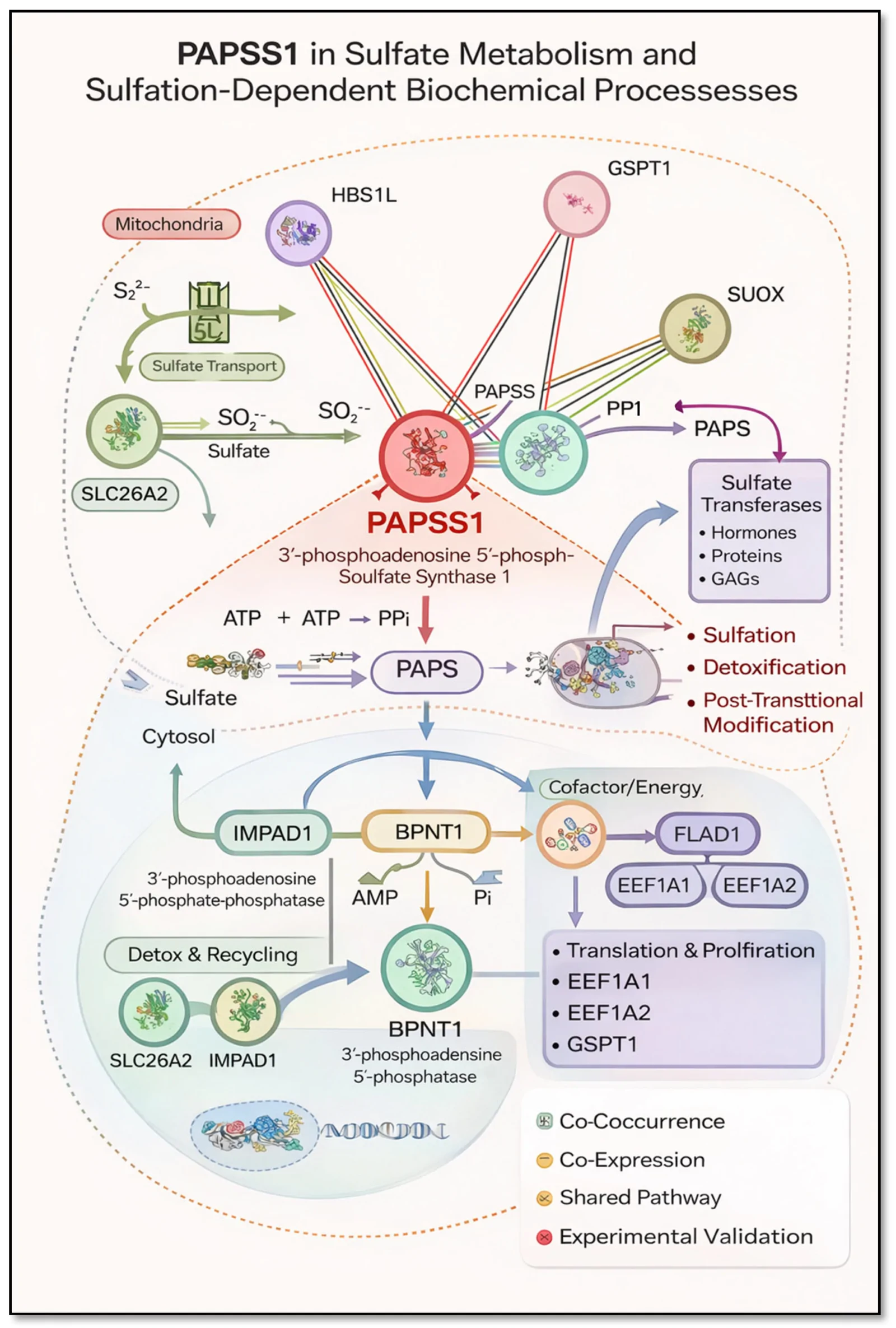
Schematic presentation of the predicted functional role of PAPSS1 within sulfate metabolism and its associated protein interaction network. PAPSS1 catalyzes the synthesis of PAPS. Upstream sulfate sully is mediated by SLC26A2. Downstream, PAPS is utilized by sulfotransferases for the sulfation of hormones and proteins, contributing to post-translational modification pathways. The accumulation of PAP (sulfation byproduct) is regulated by BPNT1 and IMPAD1. Additional interaction with FLAD1, EEF1A1, EEF1A2, and GSPT1 indicates integration of PAPSS1 within broader metabolic and proliferative cellular processes. This figure was created with AI-assisted image generation (OpenAi DALL.E) and revised by the authors. This figure is derived from the STRING database-predicted and literature-supported protein–protein interaction network and does not represent experimental validation performed within the present study.

**Table 1:** Association between PAPSS1 expression and the clinicopathological characteristics in breast carcinoma.

	PAPSS1 Low	PAPSS1 High	T: Total Number *p*: *p*-Value		PAPSS1 Low	PAPSS1 High	T: Total Number *p*: *p*-Value
Age				Vascular invasion			
≤50 Year	36 (40%)	30 (50%)	T: 150 *p* = 0.231	Negative	62 (70%)	26 (45%)	T: 147 ***p* = 0.003**
>50 Year	54 (60%)	30 (50%)		Positive	27 (30%)	32 (55%)	
Size				Estrogen receptor			
≤2 cm	29 (32%)	17 (18%)	T: 150 *p* = 0.586	Negative	23 (24%)	13 (22%)	T: 150 *p* = 0.734
>2 cm	61 (68%)	43 (72%)		Positive	68 (76%)	46 (78%)	
grade				progesterone receptor			
I	13 (15%)	10 (18%)	T: 144 *p* = 0.432	Negative	35 (38%)	19 (32%)	T: 148 *p* = 0.182
II	42 (48%)	33 (58%)		Positive	55 (62%)	38 (64%)	
III	31 (36%)	15 (25%)		HER2 status			
Tumor Stage				Negative	31 (34%)	29 (49%)	T: 150 *p* = 0.192
I	18 (20%)	9 (13%)	T: 150 *p* = 0.212	HER-2 (1+)	20 (22%)	14 (22%)	
II	43 (48%)	25 (42%)		HER-2 (2+)	15 (17)	3 (5)	
III	28 (32%)	27 (45%)		HER-2 (3+)	24 (27)	14 (24)	
LN Status				Triple-negative (TN) phenotype			
Negative	35 (39)	14 (18)	T: 150 ***p* = 0.008**	Non-TN	64 (87%)	49 (88%)	T: 130 *p* = 0.865
Positive	52 (61)	49 (82)		TN	10 (13%)	7 (12%)	
PAPSS1 Expression in Tumoral Capillaries				Nuclear Pleomorphism			
0	11 (13)	1 (2%)	T: 150 ***p* < 0.0001**	1	5 (7)	6 (10)	T: 145 *p* = 0.290
1	61 (70%)	20 (34%)		2	36 (40)	25 (42)	
2	13 (15%)	20 (32%)		3	45 (50)	28 (48)	
3	2 (2)	20 (32)					
Mitotic count				Tubule formation			
1	40 (44)	39 (66)	T: 145 ***p* = 0.036 #**	1	4 (4)	3 (2)	T: 150 *p* = 0.305
2	27 (30)	10 (17)		2	27 (30)	20 (32)	
3	19 (21)	10 (17)		3	56 (61)	40 (66)	

Note: The subgroups of each clinicopathological characteristic are detailed in the left column. The right column has the T (total number of specimens assessed). Significant *p*-values are in bold font. #: This significant association should be taken cautiously because it did not withstand significant correction after multiple comparisons using the Bonferroni test.

## Data Availability

The dataset generated and analyzed during the current study are available in the repository, [figshare] with the following accession link DOI: 10.6084/m9.figshare.32227863.

## Supplementary Materials

The following supporting information can be downloaded at: https://www.mdpi.com/article/10.3390/cancers18193143/s1, Figure S1: Flow diagram showing breast carcinoma cases included in the study and availability of clinicopathological, follow-up and immunohistochemical data; A total of 221 breast carcinoma cases were identified from the pathology archive. Availability of Follow-up data and number of cases that were successfully stained with PAPSS1 are detailed in the diagram. Abbreviations: OS, overall survival; DFI, disease-free interval; DM, distant metastasis; PAPSS1, 3′-phosphoadenosine 5′-phosphosulfate synthase 1. Figure S2: Negative control of breast carcinoma tissue sections. Breast carcinoma tissue sections included in the IHC staining experiment after omission of the primary antibody (PAPSS1). Magnification in the left panel: ×200 and in the right panel: ×400 (scale is 20 μm).

## Abbreviations

The following abbreviations are used in this manuscript:

PAPSS1	3′-phosphoadenosine-5′-phosphosulfate synthase enzyme
ER	Estrogen receptor
PR	Progesterone Receptor
TN	triple negative
LN	Lymph node
VI	Vascular invasion
